# Molecular identification of *Campylobacter jejuni* and *coli* from chicken, calves and dogs to determine its potential threat on human being

**DOI:** 10.14202/vetworld.2015.1420-1423

**Published:** 2015-12-18

**Authors:** Sonuwara Begum, M. Sekar, L. Gunaseelan, Monica Gawande, G. Suganya, P. Annal Selva Malar, A. Karthikeyan

**Affiliations:** 1 Department of Veterinary Public Health and Epidemiology, Madras Veterinary College, Chennai, Tamil Nadu, India; 2 Dean, Veterinary College and Research Institute, Namakkal, Tamil Nadu Veterinary and Animal Sciences University, Chennai, Tamil Nadu, India

**Keywords:** emerging, pathogen, thermophilic *Campylobacter*, zoonotic

## Abstract

**Aim:**

*Campylobacter* is an emerging zoonotic pathogen and one of the leading cause of foodborne infection worldwide and it has been isolated from a variety of animal species. The aim of this study was to identify *Campylobacter jejuni* and *Campylobacter coli* from dogs, calves, and poultry using polymerase chain reaction (PCR).

**Methodology:**

A total of 104 number of samples comprising cloacal swab from poultry (38), a rectal swab from dogs (40), and calves (26) were collected for the isolation of thermophilic *Campylobacters* using conventional culture method. PCR was used for identification of mapA gene for *C.jejuni* and ceuE for *C.coli*.

**Results:**

The overall presence of *Campylobacter* was found to be 67(64.42%) from the samples, out of which 6 isolates belongs to *C. jejuni* species, were 5(18.51%) from chicken and 1(4.17%) from dog was recorded and about 17 isolates belongs to *C. coli* species were 9(33.33%), 6 (25%), and 1(9.09%) from chicken, dog and calves was recorded.

**Conclusion:**

Results suggested that *Campylobacter* reservoirs chicken, calves and pet dogs can play a role as the source of infection to human beings and PCR can be an ideal tool for molecular confirmation at the species level.

## Introduction

The word “*Campylobacter*” is derived from the Greek word which denotes to its morphological shape which is curved rod, spiral or “S” shaped morphology under the microscope. The thermophilic *Campylobacter* spp. like *Campylobacter jejuni, Campylobacter coli, Campylobacter lari*, and *Campylobacter upsalensis* are able to grow at 42-43°C under microaerophilic conditions (5% O_2_, 10% CO_2_, and 85% N_2_).

*Campylobacter* is one of the leading causes of foodborne diarrheal illness worldwide [1,2]. It is one of the emerging zoonotic pathogen and is responsible for more gastroenteritis cases than any other reported bacterial species in many countries [3].

Natural reservoirs of the bacteria are the gastrointestinal tract of farm and wild animals. Direct contact with carrier’s animals is found to be a possible source of infection [4]. It is frequently isolated from a variety of animal species such as poultry, cattle, pigs, sheep, pets, wild birds, and rodents [5]. It was reported that about 70.9% of the human cases were attributed to chickens, 19.3% to cattle and 8.6% to dogs [6]. Handling or consumption of undercooked or contaminated meat is considered as the significant source of human *Campylobacter* spp. infection but also other risk factors responsible for its transmission are ingestion of contaminated dairy products, water, foreign travel, and swimming in natural sources of water [7]. Large outbreaks of campylobacteriosis are rare as most cases of human illness appear to be sporadic. Difficulties in identifying the source of sporadic infections are compounded due to the widespread occurrence of these pathogens in the environment [8,9].

Molecular methods have facilitated the development of nucleic acid-based detection methods which are more rapid, sensitive and specific. Polymerase chain reaction (PCR) has been used for diagnosis which has proven to be a fast, highly discriminative and relatively simple method [10]. Virulence factors in *C. jejuni* and *C.coli* are a useful tool to assess the potential risk of poultry as a source of *Campylobacter* infection [11]. Adopting the specific gene target in routine diagnosis will help in the improved understanding of the prevalence and the epidemiology of this emerging infection.

## Materials and Methods

### Ethical approval

Prior consent of the owners was taken before collection of a rectal swab from dogs, calves and cloacal swabs from chicken. Proper ethical considerations related to handling and not to cause any injury during sampling was taken.

### Collection of samples

A total of 104 number of samples comprising cloacal swab from poultry (38), the rectal swab from dogs (40), and calves (26). The samples were collected from Department of Clinics and Post-graduate Research Institute of Animal science, Kattupakam using sterile cotton swabs (Himedia, India) and transported in an icebox to a laboratory for processing and microbiological analysis.

### Processing of samples

The samples were collected by using sterile cotton swabs (Himedia, India) and brought immediately to the laboratory for processing. The samples were put in Blood free *Campylobacter* broth base (M1318, HiMedia Pvt. Ltd., Mumbai, India) with *Campylobacter* growth supplement (HiMedia Pvt. Ltd., Mumbai, India) and incubated under microaerophilic conditions at 42°C for 24 h. A loopful of inoculum from broth was streaked into blood free *Campylobacter* selectivity agar base (M887, HiMedia Pvt. Ltd., Mumbai, India) plates and incubated for 48 h at 42°C under micro-aerophilic conditions by using internal gas generation system. This was accomplished by using equal quantity of citric acid, sodium bicarbonate and sodium borohydride which fills the jar environment with 85% N_2_, 10% CO_2_, and 5%O_2_ [12].

### Molecular confirmation by PCR

#### Extraction of DNA

Grey color, spreading type colonies with sticky nature were suspected for *Campylobacter*. Based on colony morphology suspected colonies were picked up. The DNA of *Campylobacter* spp. isolates was prepared by taking loopful of 48 h test culture in 100 µl of sterilized DNAse and RNAse free milliQ water in micro centrifuge. The samples were vortexed and heated at 95°C for 10min, cell debris was removed by centrifugation, and 3 µl of the supernatant was used as a DNA template in PCR reaction mixture.

### Oligonucleotide primers

Stock solutions of the primers were made in nuclease-free water and stored at −20°C. The working solutions were made to 10pmol/µl after suitable dilution. The isolates were identified at genus level by PCR targeting the mapA and ceuE gene of Genus *Campylobacter*. The primer used in the present study is taken from already published article [10] and the details of the primer pair used are given in (Table-1). All PCR amplifications were performed in a mixture (25 µl) containing nuclease free water, primers, Taq polymerase, and template DNA. The amplification was carried out in a thermal cycler with the following cycling conditions as shown in (Table-2).

**Table-1:** Sequence of primers used in PCR for *C. jejuni* and *C. coli*.

Primer name	Sequence	Product size
MDmapA1FP	5’-CTA TTT TAT TTT TGA GTG CTT GTG3-’	589 bp
MDmapA2RP	5’-GCT TTA TTT GCC ATT TGT TTT ATT A3-’	
COL3 F	5’-AAT TGA AAA TTG CTC CAA CTA TG3-’	462 bp
MDCOL2 R	5’-TGA TTT TAT TAT TTG TAG CAG CG3-’	

*C. jejuni=Campylobacter jejuni, C. coli=Campylobacter coli*, PCR=Polymerase chain reaction

**Table-2:** Cycling conditions for the PCR assay.

Step	Temperature	Time	Cycle
Initial denaturation	95°C	10 min	
Denaturation	95°C	30 sec	35 cycles
Primer annealing	59°C	60 sec	
Extension	72°C	60 sec	
Final extension	72°C	10 min	

PCR=Polymerase chain reaction

## Results

### Prevalence of Campylobacter

In the present study, the overall prevalence of *Campylobacter* was found to be 67 (64.42%) from the samples based on colony morphology and microscopic examination, out of which species wise prevalence was 24(60%) from dog, 27(71.05 %) from poultry and 16(61.54%) from calves was recorded.

### Molecular identification of the isolates

**Figure-1:**
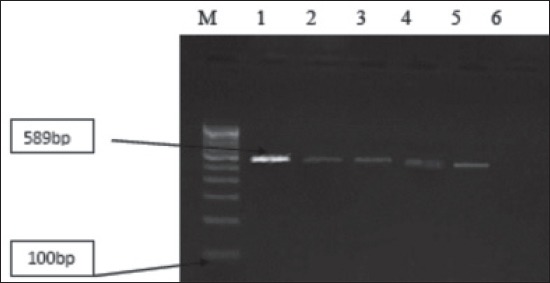
Agarose gel showing polymerase chain reaction amplified product at (589 bp) for mapA genes in Campylobacter isolates. M: Molecular marker (100 bp, Fermentas); Lane 1: Positive control; Lane 2-5: Positive samples; Lane 6: Negative control

**Figure-2:**
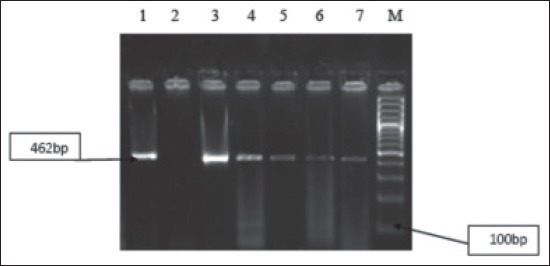
Agarose gel showing polymerase chain reaction amplified product at (462 bp) for ceu genes in Campylobacter isolates. Lane 1: Positive control; Lane 2: Negative control; Lane 3-7: Positive samples; M: Molecular marker (100 bp, Fermentas).

All the *Campylobacter* isolates recovered from conventional culture method was tested by PCR targeting mapA gene for *C.jejuni* and ceuE for *C.coli*. Out of the 67 *Campylobacter* isolates 6 isolates belong to *C.jejuni* species, were 5 (18.51%) from poultry and 1 (4.17%) from dog and 17 to *C.coli* species were 9 (33.33%) from poultry, 6 (25%) from dog and 1 from calves were identified as zoonotic thermophilic *Campylobacter* (Figures-1 and -2).

## Discussion

Thermophilic *Campylobacters* are major foodborne pathogens of animal origin and leading cause of gastrointestinal infections. The importance of this disease is compounded by the fact that very low doses of *Campylobacter* in the food samples are capable of causing infections unlike other foodborne infection. In India, the prevalence of *Campylobacter* is underestimated which may be due to its way of cooking and food habit.

The advent of molecular biology techniques such as PCR has largely replaced the conventional isolation procedure by virtue of its speed, sensitivity and discriminating power. The conventional isolation procedure although time-consuming and cumbersome still remains the gold standard.

The finding of the present study is comparable with a report of Kassa *et al*. [13] were the prevalence of *Campylobacter* spp. in chickens observed was (68.1%). In one study, the frequency of isolation of *Campylobacter* spp. in dogs varied from 17% (Brazil, Argentina) to 76.2% (Denmark) [4,14]. However, there are several reports of higher isolation rates of *Campylobacter* from cattle, ranging from 5% to 89.4% [15]. Workman *et al*. [16] also reported the prevalence of *Campylobacter* spp. from rectal swabs of dogs to be 46.9% and from cloacal swabs of broiler chicks 94.2%. Amar *et al*. [17] reported the prevalence of *Campylobacter* in chicken flocks (33-44%), cattle (15%) and in healthy dogs (6%) in Switzerland.

Molecular characterization of two different gene targets for *C. jejuni* and *C. coli* was attempted; the targeted gene was mapA and ceuE. In one study, Sandberg *et al*. [18] reported that about 7% of the isolates from dog were positive for *C.coli* and 6% for *C.jejuni*. A Dutch study reported the prevalence ranging from 20% to 31% in poultry were *C. jejuni* was (5.38%) and *C.coli* (2.3%) [19,20]. Awadallah *et al*. [21] also reported *C.coli* (7.4%) and *C. jejuni* (3.7%) from chicken cloacal swabs.

Prevalence of *Campylobacter* varies between countries depending on the level of hygienic measures followed. This variation in getting a low number of isolates positive for mapA and ceuE gene may be due to the primers used, the prevalence of inhibitors, laboratory condition, seasonal variations as well as geographic diversity in the distribution of *C.jejuni* and *C.coli* isolates.

## Conclusion

Our findings suggest that chicken, calves and pet dogs can play a role as the reservoir of potentially pathogenic *Campylobacter* strains for humans. Hence, proper public health protection measures should be taken to control it and prevent its transmission from the reservoir. PCR can be used an ideal tool for molecular confirmation at the species level.

## Author’s Contributions

MS and LG have designed the study project as well as corrected the manuscript, SB has done the research work, data compiling and manuscript preparation. MG, GS, PASM, and AK helped in doing the research work. All authors read and approved the final manuscript.
